# Phase contrast cardiac magnetic resonance imaging versus transoesophageal echocardiography for the evaluation of feasibility for transcatheter closure of atrial septal defects

**DOI:** 10.1186/s43044-022-00269-7

**Published:** 2022-04-13

**Authors:** Tariq Rashid Shah, Jahangir Rashid Beig, Naseer Ahmad Choh, Fayaz Ahmad Rather, Irfan Yaqoob, Vicar Mohammad Jan

**Affiliations:** 1 Department of Cardiology, SKIMS, Srinagar, J&K India; 2 Department of Cardiology, Super Speciality Hospital, Government Medical College, Srinagar, Jammu and Kashmir 190010 India; 3 Department of Radiology, SKIMS, Srinagar, J&K India

**Keywords:** Atrial septal defect, Cardiac magnetic resonance imaging, Transoesophageal echocardiography, Transcatheter closure

## Abstract

**Background:**

This prospective study was aimed at comparing phase contrast cardiac magnetic resonance imaging (PC-CMR) with 2D transoesophageal echocardiography (TEE) for determining potential candidature for transcatheter closure in ostium secundum ASD (OS-ASD) patients. We included consecutive adult patients with OS-ASD for the evaluation of feasibility for transcatheter closure using 2D-TEE and PC-CMR over a period of 2 years. Patients who fulfilled the conventional criteria for transcatheter closure, i.e. maximum ASD diameter ≤ 34 mm, adequate rims (≥ 5 mm, except for anterosuperior rim), and normal pulmonary venous drainage on both imaging modalities, were taken for device closure. In patients where there was discrepancy in the measurements of ASD diameter or rim size, making them eligible for device closure on one imaging modality and ineligible on the other hand, provisional device closure was attempted. All patients who underwent transcatheter closure were followed up to 6 months to rule out any complications.

**Results:**

A total of 58 patients (mean age 35.93 ± 10.59 years) were enrolled in the study. Overall, there was significant positive correlation between 2D-TEE and CMR measurements of maximal ASD diameter and rim size (*p* < 0.001). However, TEE significantly underestimated maximal ASD diameter and posteroinferior rim size in comparison with CMR (*p* = 0.013 and *p* = 0.023, respectively). 46 (79.3%) patients were suitable for transcatheter closure on CMR, while 44 (75.9%) were eligible on TEE. Transcatheter closure was attempted in 48 patients based on imaging findings and was successful in 46 (95.8%) patients. Device closure was unsuccessful in 2 patients with defect size < 34 mm on TEE but > 34 mm on CMR. Among 7 patients with deficient posteroinferior rim on TEE, 5 had sufficient rim on CMR and underwent successful transcatheter closure. CMR detected anomalous pulmonary venous drainage in one patient which was missed on TEE, hence excluding the patient from transcatheter closure. Mean device size was 28.3 ± 7.4 mm and correlated more strongly with the defect dimensions on PC-CMR (*r* = 0.85, *p* < 0.001) compared to TEE (*r* = 0.71, *p* = 0.02).

**Conclusions:**

PC-CMR may to be superior to 2D-TEE for the preprocedural planning and feasibility assessment for transcatheter closure in adult patients with ostium secundum ASD.

## Background

Atrial septal defect (ASD) is one of the most common forms of congenital heart diseases (CHD), accounting for 6–10% of all CHD cases and 30–40% of adult CHD patients [1]. Ostium secundum ASD is the most frequent type of ASD and comprises 70% of all such cases. First described by King et al. in 1976, transcatheter closure has become the procedure of choice for the treatment of secundum ASD patients with suitable morphology who are symptomatic or have hemodynamically significant shunts [2, 3]. Minimally invasive nature, shorter hospital stays, fewer complications, and greater cost effectiveness are the unequivocal advantages of this technique over conventional surgical closure [3, 4]. Meticulous preprocedural morphological evaluation for the feasibility of device closure forms the cornerstone of achieving successful results with this procedure [5, 6].

Transoesophageal echocardiography (TEE) is the most validated method of evaluating the feasibility of device closure in ASD patients [5–7]. Except for some paediatric patients with excellent acoustic windows where transthoracic echocardiography (TTE) may alone be sufficient for management decisions, TEE is recommended in all patients before contemplating transcatheter closure for detailed assessment of type, size, shape, number of defects, presence of adequate rims for device anchorage, and ruling out any associated anomalies that could potentially complicate or preclude device closure [7–9].

Cardiac magnetic resonance imaging (CMR) has dramatically evolved as an imaging modality for congenital heart disease over the last couple of decades [10]. It offers distinct advantages including superb spatial and temporal resolution, large field of view not restricted by body habitus or acoustic window, lesser operator dependence, and three-dimensional multiplanar imaging capability of various cardiac and extracardiac structures. Furthermore, CMR is a versatile investigative modality providing highly accurate and reproducible information about cardiovascular anatomy and physiology [11]. Phase contrast imaging represents a recent advance in CMR that relies on motion-induced phase shifts for the measurement of local flow velocities within cardiac or vascular structures [12, 13]. In patients with ASD, phase contrast CMR (PC-CMR) allows 3D *en face* visualisation of the defect and direct quantification of the left to right shunt [14, 15]. To date, there is limited data published in the literature where PC-CMR has been compared to TEE for the evaluation of feasibility for device closure in ASD patients [14–20]. The purpose of this study was to compare PC-CMR with 2D-TEE in secundum ASD patients for comprehensive morphological evaluation and determining potential candidature for transcatheter closure.

## Methods

The present study was a prospective and comparative study conducted in the Department of Cardiology, Sher-i-Kashmir Institute of Medical sciences over a period of 2 years. We included consecutive adult patients (18 years or older) diagnosed with ostium secundum ASD on TTE for the evaluation of feasibility for transcatheter closure using 2D-TEE and PC-CMR. Patients with contraindication for CMR, other types of ASD, i.e. ostium primum or sinus venosus defects, atrial septal aneurysm (ASA), or multiple ASDs, were excluded from the study. An informed consent was obtained from each patient before enrolment in the study and the study protocol was approved by the Institutional Ethics Committee. All the eligible patients underwent evaluation by both PC-CMR and 2D-TEE within 2 weeks of initial diagnosis by TTE.

### Transoesophageal echocardiography

2D-TEE of each patient was performed by a team of two experienced cardiologists who were blinded to the CMR details of the patient. After sedating the patient with 2–5 mg midazolam, the procedure was performed using Aloka, Prosound, SSD α-110 (South Korea) 2D TEE system with multiplanar 5 MHz transducer, while continuously recording a 1-lead ECG. The inter-atrial septum was visualised after intra-oesophageal transducer placement at different rotation angles (0°–130°) at the upper oesophageal, mid-oesophageal, and gastroesophageal junction levels. Maximum ASD size was measured at ventricular end-systole. Atrial septal margins were measured as per conventional definitions [7, 9]. The anterior inferior (AI) rim was measured from the defect to the mitral valve, the anterior superior (AS) rim from the defect to the aortic root, posterior inferior (PI) rim from the defect to the inferior vena cava and posterior superior (PS) rim from the defect to the superior vena cava. The TEE views for measurement of the rims were: the mid-oesophageal four-chamber view for AI rim, basal short axis view for AS rim, and biatrial views for PI and PS rims. All the rims were evaluated in at least three sequential related multiplane views in 15° increments. For the better visualisation of posteroinferior rim the TEE probe was advanced into the stomach, retroflexed, and slowly withdrawn to lower oesophagus, while imaging in the long axis at 90° ± 20°. This manoeuvre allows the ultrasound beam to be directed perpendicular to the PI rim thus profiling it more clearly. For the imaging of right pulmonary veins, the probe was placed at mid-oesophageal level at 45° ± 10°, rotated clockwise and gradually withdrawn till veins were visualised. Left pulmonary veins were visualised by placing the probe at mid-oesophageal level at 120° ± 10°, rotating counterclockwise with gradual withdrawal. Additional colour Doppler signal imaging (colour scale 35–40 cm/s) was used to outline the margins of the defect. All measurements were based on consensus opinion of both cardiologists.

### Cardiac magnetic resonance imaging

CMR of each patient was performed by a team of two experienced radiologists who were blinded to the TEE details of the patient. All examinations were performed with a 1.5 T whole-body MR imaging unit (Magnetom Avanto, Siemens Medical Systems, Germany). The maximum gradient performance of this system was 45 mT/m amplitude with slew rate of 200 T/m/ms. A five-element cardiac phased-array coil was used for signal acquisition. After taking a HASTE localiser, 4 chamber views were obtained using a retrospectively gated cine MRI sequence (trufi/FLASH) during end-expiratory breath hold of 8–10 s. After getting a clear view of the ASD in 4 chamber planes, biatrial parasagittal cine sections were obtained through the defect with a section thickness of 6 mm and no intersection gap. Through-plane phase contrast sequences were planned on the 4 chamber and biatrial planes using an encoding velocity (*V*_enc_) of 60–80 cm/s, to ensure sensitivity to lower blood flow velocities close to the defect edge, thereby preventing underestimation of the diameter of large defects with low shunt flow velocities. One to three contiguous cine phase-contrast MR imaging sections were obtained in the atrial septal plane to provide an *en face* view of the spatial position of the ASD. From this projection, sections were obtained from the defect toward the SVC, the IVC, the aortic root, and the atrioventricular valves. Care was taken to match the TEE protocol as closely as possible. Maximal ASD diameter was assessed at the ventricular end-systole and the sizes of the four ASD rims (described above) were measured. Size measurements obtained from *en face* images were only accepted if the defect was truly located in plane, as suggested from a narrow rim of signal void in the magnitude images. Pulmonary and systemic venous return was assessed with two-dimensional time-of-flight MR angiography with transverse-plane acquisitions. To enhance signal from thoracic veins, a trigger delay was chosen so that images were acquired during early diastole, when venous flow is maximal. All measurements were based on consensus opinion of both radiologists.

### Transcatheter device closure

Percutaneous device closure was performed by a team of two experienced interventional cardiologists. Patients who fulfilled the conventional criteria for transcatheter device closure, i.e. maximum ASD diameter ≤ 34 mm, adequate rims (≥ 5 mm, except for AS rim), and normal pulmonary venous drainage on both imaging modalities were taken for device closure [8]. Patients in whom there was discrepancy in the measurements of ASD diameter or rim size which made them eligible for device closure on one imaging modality and ineligible on the other hand were provisionally taken up for the procedure and device closure was attempted. All the defects were closed using an Amplatzer Septal Occluder (St. JudeMedical, St. Paul, MN, USA) under fluoroscopic and 2D-TEE guidance using standard techniques. The device size was chosen depending on the maximum ASD diameter and adequacy/floppiness of the rims. Procedural success was defined by standard criteria, i.e. stable device position after release from the delivery cable, absence of any residual shunt across the defect on colour flow imaging, and no impingement of important structures like ostia of coronary sinus or venae cavae, and mitral or tricuspid valves on post-procedural TEE [3, 4].

### Follow-up

All the patients were observed for 24–48 h in the hospital for any acute complications (device embolisation, stroke/transient ischemic attack, pericardial effusion, etc.) and discharged after confirming satisfactory results on repeat TTE as per hospital protocol. Each patient received dual antiplatelet therapy (aspirin 75 mg and clopidogrel 75 mg per day), starting 24 h before the procedure and continued 1 month after the procedure. Aspirin monotherapy was continued for another 5 months. All patients were followed up at 1-week, 1-month, 3-month, and 6-month intervals on out-patient basis with clinical history taking and cardiovascular examination. A repeat 2D-TEE was performed at 6 months to rule out any late complications, i.e. device erosion.

### Statistical analysis

Statistical analysis was performed by SPSS software package (version 20.0, SPSS Inc, Chicago, Illinois, USA). All continuous variables were expressed as mean ± standard deviation (SD), and categorical variables were reported as frequency and percentages. Pearson’s correlation coefficients were used to assess the strength of relationship between measurements of defect and rim sizes on TEE and CMR. Differences in estimations were calculated using paired *t*-tests and expressed as mean difference (95% confidence interval). Bland–Altman comparative analysis was performed to demonstrate agreement between the TEE and CMR measurements. Statistical significance was defined as a *p* value of < 0.05.

## Results

A total of 65 patients were initially screened for eligibility by TTE over a period of 2 years. Five patients were excluded from the study based on TTE findings (three patients with multiple ASDs and two with ASA). One patient had claustrophobia and another did not give consent for the study. Finally, 58 patients who fulfilled the eligibility criteria were enrolled in the study. The mean age of our patients was 35.93 ± 10.59 years (range 18–60 years). The majority (73%) of patients were in the age group of 18–40 years. Female patients outnumbered males in the present study [42 (72.4%) vs. 16 (27.6%)]. All the patients in the present study were in sinus rhythm.

**Fig. 1:**
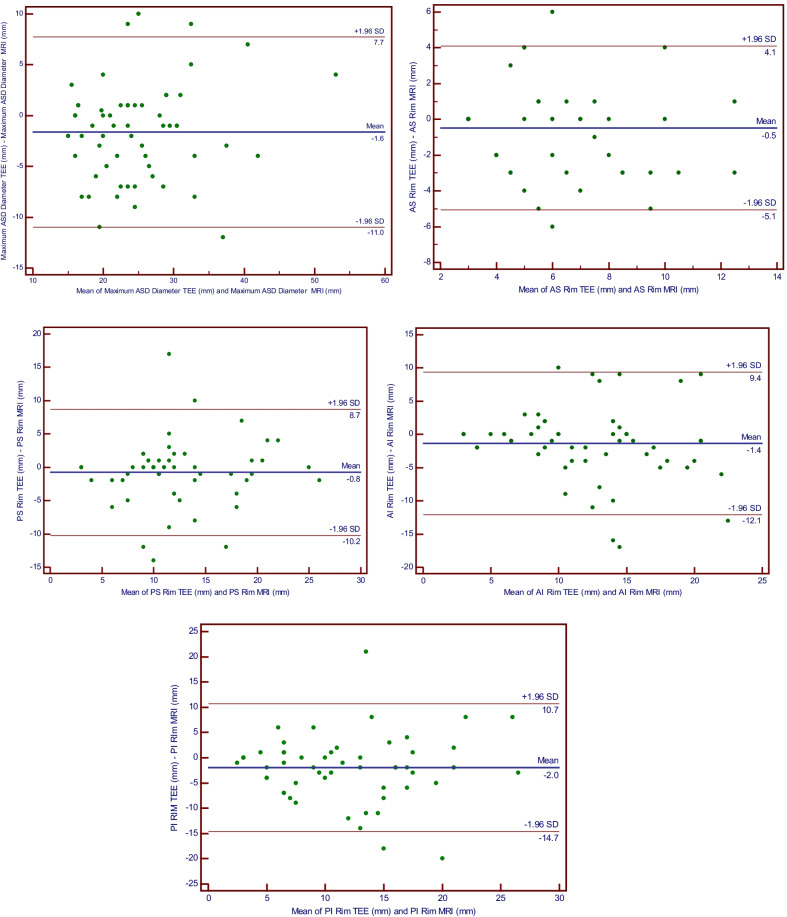
Bland Altman plot for agreement of modes of measurement between TEE and CMR for Maximum ASD diameter and rim sizes. Note: ASD, Atrial Septal Defect; TEE, Transesophageal Echocardiography; MRI, Magnetic Resonance Imaging; AS, Anterosuperior; PS, Posterosuperior; AI, Anteroinferior; PI, Posteroinferior

**Fig. 2:**
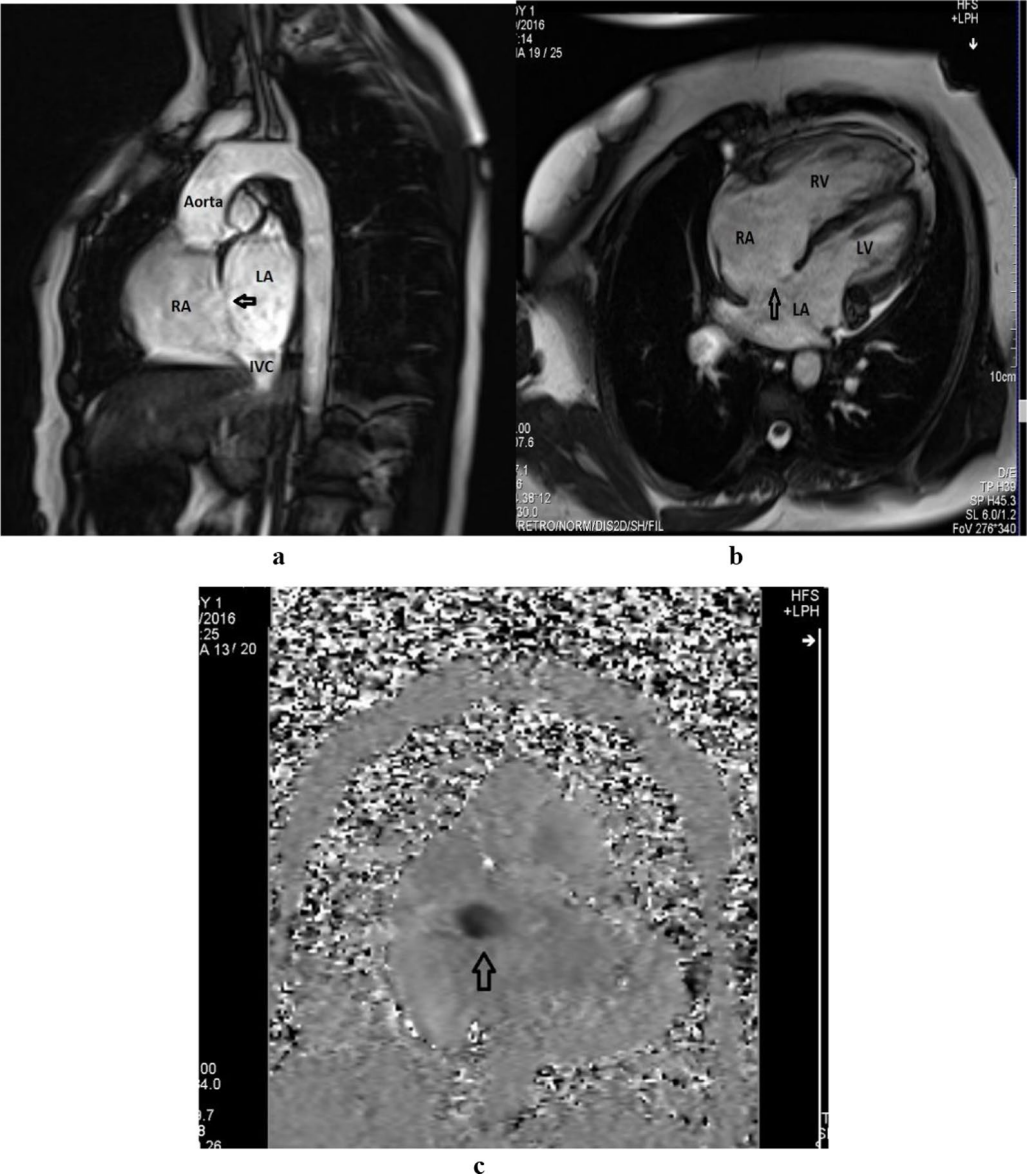
Phase contrast CMR images depicting ASD (arrow heads) in Oblique Sagittal view (**a**), Axial/Four chamber view (**b**), and *enface* view (**c**). Note: ASD, Atrial Septal Defect; CMR, Cardiac Magnetic Resonance Imaging

Correlation between defect diameter and rim sizes on 2D-TEE and CMR are described in Tables 1 and 2. On Pearson correlation analysis there was significant positive correlation between TEE and CMR measurements of maximal ASD diameter, AS rim, PS rim, AI rim, and PI rims (*p* < 0.001). Paired *t*-tests demonstrated that maximal ASD diameter and PI rim size were consistently smaller on TEE in comparison with CMR (*p* = 0.013 and *p* = 0.023, respectively). There was no significant difference between mean measurements of other rims on either modality. Bland–Altman analysis revealed an overall good agreement between 2D-TEE and CMR measurements of ASD diameter (Fig. 1) and rim sizes (Fig. 2).

**Table 1:** Pearson correlation analysis of ASD diameter and rim sizes between TEE and CMR

	Parameter	*N*	Correlation (*r*)	*p* value
Pair 1	ASD diameter TEE and ASD diameter CMR	58	0.81	< 0.001
Pair 2	AS rim TEE and AS rim CMR	58	0.68	< 0.001
Pair 3	PS rim TEE and PS rim CMR	58	0.64	< 0.001
Pair 4	AI rim TEE and AI rim CMR	58	0.54	< 0.001
Pair 5	PI rim TEE and PI rim CMR	58	0.56	< 0.001

*ASD* atrial septal defect, *TEE* transoesophageal echocardiography, *CMR* cardiac magnetic resonance imaging, *AS* anterosuperior, *PS* posterosuperior, *AI* anteroinferior, *PI* posteroinferior, *N* number

**Table 2:** Paired t-test analysis of ASD diameter and rim sizes between TEE and CMR

Parameter	TEE (mean ± SD)	CMR (mean ± SD)	Mean difference (95% CI)	*p* value
ASD diameter (mm)	24.16 ± 9.09	25.77 ± 7.47	− 1.6 (− 2.8, − 0.4)	0.013
AS rim (mm)	5.34 ± 2.61	5.82 ± 3.02	− 0.5 (− 1.1, 0.1)	0.112
PS rim (mm)	11.81 ± 5.87	12.59 ± 5.52	− 0.8 (− 2.0, 0.5)	0.225
AI rim (mm)	11.58 ± 5.33	12.97 ± 6.09	− 1.4 (− 2.8, 0.1)	0.060
PI rim (mm)	10.81 ± 6.80	12.79 ± 6.93	− 2.0 (− 3.7, − 0.3)	0.023

*ASD* atrial septal defect, *TEE* transoesophageal echocardiography, *CMR* cardiac magnetic resonance imaging, *AS* anterosuperior, *PS* posterosuperior, *AI* anteroinferior, *PI* posteroinferior

Based on the conventional criteria for device closure, 46 (79.3%) patients were suitable for transcatheter closure on CMR, while 44 (75.9%) were eligible on TEE. Six patients had maximum defect size > 34 mm on CMR. Four of these were excluded from device closure as defect size on TEE was also > 34 mm. The other two (TEE defect size 28 mm and 30 mm vs. CMR defect size 35 mm and 37 mm, respectively) were provisionally taken up for transcatheter closure, but the device could not be deployed as the largest available Amplatzer ASO device did not achieve a stable position due to insufficient rim support. Seven patients had PI rim < 5 mm on TEE. Two of these were excluded from transcatheter closure as CMR also showed a deficient PI rim (< 5 mm). The other five had > 5 mm PI rim on CMR and thus were provisionally scheduled for device closure. In all of these patients, the procedure was completed successfully with total occlusion of the defect using an appropriately sized ASO device, which was released only after confirming a stable position by ‘Minnesota wiggle manoeuvre’ and ruling out impingement of important structures (especially protrusion into IVC hampering inflow to the right atrium). Two patients had deficient PS rim and one had deficient AI rim on both CMR and TEE making them ineligible for the procedure. CMR detected anomalous drainage of left upper pulmonary vein into innominate vein in one patient which was missed on 2D-TEE, hence excluding the patient from transcatheter closure.

Transcatheter closure was attempted in 48 patients based on imaging findings, and a device could be successfully deployed in 46 (95.8%). Mean device size was 28.3 ± 7.4 mm and correlated more strongly with the defect dimensions on PC-CMR (*r* = 0.85, *p* < 0.001) as compared to 2D-TEE (*r* = 0.71, *p* = 0.02). None of the patients who underwent device closure had any complications on follow-up over a period of 6 months.

## Discussion

The present study demonstrates that PC-CMR-derived measurements of ASD diameter and rim sizes show strong positive correlation with the corresponding values assessed by conventional 2D TEE in adult patents with ostium secundum ASD. PC-CMR also appears to be superior to 2D-TEE for evaluating the preprocedural feasibility for device closure in these patients, as 2D-TEE significantly underestimates maximal ASD diameter and PI rim size and can occasionally miss an anomalously draining pulmonary vein. In this study, all the patients deemed to be suitable for device closure on CMR ultimately underwent a successful transcatheter closure with a device whose size correlated strongly with the defect size measured on CMR.

TEE is the most widely available and validated method for evaluating the feasibility for device closure in adult ASD patients and is recommended for all patients who are being planned for this procedure [8, 9]. It provides a thorough insight into the defect morphology including type, size, shape, number of defects, presence of adequate rims for device anchorage, and ruling out any associated anomalies that could potentially complicate or preclude device closure [5–9]. TEE also serves as an invaluable tool for intra-procedural guidance and allows safe and accurate device deployment by ensuring stable device position, ruling out residual shunts and impingement of important structures before device release [3, 4, 21]. However, TEE is a semi-invasive imaging modality causing discomfort to the patients and requires sedation or occasional administration of general anaesthesia in uncooperative patients. Furthermore, although a relatively safe procedure, serious complications including oesophageal laceration or perforation, upper GI bleeding, pharyngeal tears, cardiac arrhythmias, laryngospasm, and hypoxia have been described in 0.1–0.9%. Mortality is rare, occurring in 0.01–0.02% cases [22, 23].

2D TEE is the most widely used mode for assessing ASD anatomy before transcatheter closure. Given the two dimensional nature, it is fraught with inherent limitations of comprehensively defining the shape, eccentricity and multiplicity of the defects, especially in complex secundum defects [24]. With the advent of real-time 3D matrix array TEE transducers in recent years, many of these limitations have been circumvented. Real-time 3D TEE allows more comprehensive interrogation of atrial septal anatomy and dynamic *en face* visualisation of ASD throughout the cardiac cycle. However, limited availability and lack of widespread expertise are the major stumbling blocks in its routine use in our part of the world. Furthermore, the posteroinferior part of the atrial septum may be inadequately visualised due to artefactual dropout, small fenestrations may escape detection due to limited spatial resolution, and patient movement during image acquisition can result in malalignment and reconstruction artefacts [25, 26].

Besides the obvious advantages of three-dimensional imaging and non-invasive nature, CMR generally outperforms TEE in terms of larger field of view, unlimited tomographic planes acquisition, and lesser operator dependence [10, 11]. Conventional CMR sequences including spin-echo technique and cine gradient-echo technique are often inaccurate in depicting the size and margins of secundum ASD, owing to septal thinning adjacent to the defect and low interatrial pressure gradient across the defect [16]. PC-CMR imaging, on the other hand, has been demonstrated to be superior to these techniques in reliably defining the size and shape of ASDs as it more sensitively detects localised low velocity phase shifts across the defect and provides a 3D *en face* view of the defect based on flow related signal enhancement [15–20]. In a study of 30 adult patients, Holmvang et al. demonstrated that ASD dimensions measured on PC-CMR showed an excellent correlation with balloon sizing of the defect during catheterisation as well as template standards measured during surgery. Spin-echo imaging overestimated the defect diameter by 48% in the same study that was attributed to signal dropout in the fossa ovalis region due to septal thinning [16]. Beerbaum et al. in a study of 65 paediatric patients, demonstrated good agreement between PC-CMR and TEE-derived ASD size (mean difference < 1 mm). Among 30 patients who were scheduled for transcatheter closure based on PC-CMR findings, 5 patients were found to have unexpectedly large defects on stretched balloon sizing and hence referred for surgery [17]. Consistent with the previous studies, there was a strong correlation between TEE and CMR *vis a vis* ASD diameter in the present study (*r* = 0.81, *p* < 0.001). However, TEE significantly underestimated the defect size compared to PC-CMR (mean difference − 1.6 mm; 95% CI − 2.8, − 0.4 mm). Furthermore, 2 patients with defect diameter < 34 mm on TEE versus > 34 mm on CMR could not undergo successful transcatheter closure as the largest available device size (40 mm) did not suffice in completely occluding the defect while achieving a stable position before release. We did not use balloon sizing technique in this study as contemporary data suggest that it is no longer necessary and may occasionally lead to device oversizing [27, 28]. Our results also demonstrated that the final device size that successfully occluded the defect correlated more strongly with the defect dimensions on PC-CMR (*r* = 0.85, *p* < 0.001) as compared to TEE (*r* = 0.71, *p* = 0.02). These findings are in sync with those published by Thomson et al. and Durongpisitkul et al. which demonstrated better correlation between device size and ASD diameter on CMR in comparison with intracardiac echocardiography (ICE) and TEE, respectively. [15, 20] The ability to visualise the defect *en face* on PC-CMR provides an unequivocal advantage over conventional echocardiography in clearly outlining the defect especially when the location or shape is eccentric.

In so far as the assessment of various rims is concerned, we found a significant correlation between rim sizes measured on TEE and CMR (*r* = 0.68, *p* < 0.001 for AS rim; *r* = 0.64, *p* < 0.001 for PS rim; *r* = 0.54, *p* < 0.001 for AI rim; *r* = 0.56, *p* < 0.001 for PI rim). The mean difference of rim measurements was less than 1 mm for all rims except for PI rim. TEE significantly underestimated PI rim size compared to CMR (mean difference − 1.98; 95% CI − 3.68, − 0.28; *p* = 0.023). This was even after we used all the recommended TEE manoeuvres including retroflexion and withdrawal of the probe from stomach for adequate visualisation of PI rim. Among seven patients with insufficient PI rim on TEE, five had rim size > 5 mm on CMR and subsequently underwent a successful device closure. There are a few studies which have demonstrated that transcatheter closure, although difficult, may be safe and feasible in some patients with deficient PI rim on TEE [29, 30]. Given the facts that inadequate evaluation of the posteroinferior part of the atrial septum represents an inherent limitation of TEE, even with the latest 3D technology, and more than 70% patients with deficient PI rim on TEE actually had sufficient rim on CMR in our study, we can infer that CMR is superior to TEE in deciding the candidature for device closure based on PI rim size [13, 24–26]. In the present study, CMR picked up anomalous drainage of left upper pulmonary vein into innominate vein in one patient which was missed on TEE, hence excluding the patient from transcatheter closure. Previous studies have shown that CMR may identify additional cardiac or extracardiac anomalies in up to 20% patients that alter clinical management decisions [15, 17]. Meticulous echocardiographic evaluation and exclusion of patients with multiple defects or atrial septal aneurysms could have attributed to such lower proportion in the present study. Besides the lack of widespread availability, there are certain important limitations to CMR that need to be mentioned. Apart from prolonged scanning times and ability to hold breath, CMR has been shown to be inferior to TEE in detecting patent foramen ovale or small septal fenestrations [15, 31]. Furthermore, PC-CMR imaging does not reliably depict septal thickness because the method depends on flow imaging rather than on structural delineation of thin membranes. Therefore, “floppy” septa, which are often found to require a large closure device after balloon-sizing in the catheter laboratory, can sometimes be missed at PC-CMR imaging [17]. Finally, the utility of CMR is limited by the presence of metallic prostheses or claustrophobia in a small percentage of patients.

## Limitations

There are some important limitations to the present study. First, this was a small single-centre study with limited sample size that included only adult patients. Therefore, extrapolation of these results to broader patient population would require validation from larger adequately powered multicentre studies including paediatric patients. Second, all the patients in this study were in sinus rhythm and had regular breathing patterns. Hence, our results do not apply to patients with arrhythmias or irregular breathing patterns in whom image quality may be distorted by motion artefacts. Third, we excluded patients with atrial septal aneurysms, multiple ASDs and multifenestrated ASDs from this study. Hence, we cannot comment on the utility and accuracy of CMR in these patient subgroups. Fourth, we did not use balloon sizing of the defect during cardiac catheterisation for comparison with TEE- or CMR-derived defect dimensions. The device size chosen for defect occlusion was based on operator’s discretion after integrated assessment of TEE and CMR results. Therefore, stronger correlation between CMR-derived defect dimensions and final device size could partly be a result of selection bias. Lastly, we did not use real-time 3D TEE in our study due to its non-availability at our centre. Whether the superiority of CMR persists despite using 3D TEE technology needs to be demonstrated in future studies.

## Conclusions

PC-CMR may be superior to 2D-TEE for the preprocedural planning and feasibility assessment for device closure in adult patients with ostium secundum ASD. 2D-TEE significantly underestimates maximal ASD diameter and PI rim size as compared to CMR and can occasionally miss coexistent cardiac or extracardiac anomalies that may influence clinical management decisions. Whether the superiority of CMR persists despite using the latest real-

time 3D TEE technology needs to be demonstrated in future studies.

## Data Availability

The datasets used and/or analysed during the current study are available from the corresponding author on reasonable request.

## Abbreviations

OS-ASD: Ostium secundum atrial septal defect
PC-CMR: Phase contrast cardiac magnetic resonance imaging
TEE: Transoesophageal echocardiography
